# Clinical and Humoral Immune Features of Post-COVID Syndrome One Year After SARS-CoV-2 Infection in Elderly Patients with Type 2 Diabetes

**DOI:** 10.3390/v18060671

**Published:** 2026-06-14

**Authors:** Svetlana Bolshakova, Saule Altynbekova, Zhangentkhan Abylaiuly, Gulim Aldangarova

**Affiliations:** 1 Department of Endocrinology, Asfendiyarov Kazakh National Medical University, Almaty 050000, Kazakhstan; 2 Department of Postgraduate Education, Kazakh–Russian Medical University, Almaty 050004, Kazakhstan

**Keywords:** post-COVID syndrome, type 2 diabetes mellitus, older adults, COVID-19, immune response, metabolic disturbances

## Abstract

**Background:** Post-COVID syndrome represents a significant medical and public health challenge, particularly among older adults and individuals with type 2 diabetes mellitus (T2DM), in whom disturbances in immune and metabolic homeostasis may contribute to the development and persistence of symptoms following SARS-CoV-2 infection. **Objective:** To investigate the clinical, immunological, and metabolic characteristics of post-COVID syndrome in older adults with T2DM. **Methods:** A cross-sectional comparative study was conducted involving 141 patients aged ≥ 60 years who were evaluated more than one year after SARS-CoV-2 infection. Clinical data, anthropometric measurements, complete blood count parameters, biochemical markers, glycated hemoglobin (HbA1c), and SARS-CoV-2-specific IgG antibodies were assessed. Statistical analyses were performed using nonparametric methods, while Pearson’s χ^2^ test was applied for categorical variables. A *p*-value < 0.05 was considered statistically significant. **Results:** Symptoms consistent with post-COVID syndrome one year after SARS-CoV-2 infection were identified in 53.2% of participants. No significant differences in anthropometric characteristics, hematological parameters, or most biochemical markers were observed between patients with and without post-COVID syndrome. Patients with T2DM exhibited higher fasting glucose, HbA1c, and SARS-CoV-2–specific IgG antibody levels, reflecting underlying metabolic characteristics and differences in humoral immune responses during the late post-COVID period. **Conclusions:** Post-COVID syndrome symptoms were frequently observed among older adults at the time of assessment, more than one year after SARS-CoV-2 infection, despite normalization of most laboratory parameters. In patients with T2DM, higher glucose, HbA1c, and antibody levels likely reflect underlying metabolic characteristics rather than a direct effect of post-COVID syndrome. Further longitudinal studies are warranted to clarify the long-term clinical significance of the observed metabolic and immunological findings.

## 1. Introduction

The long-term consequences of coronavirus disease 2019 (COVID-19) represent an important interdisciplinary clinical challenge and are collectively referred to as post-COVID syndrome. This condition encompasses a broad spectrum of clinical manifestations, ranging from mild fatigue to significant organ dysfunction, that may persist for weeks or months following the acute phase of SARS-CoV-2 infection [1].

According to published meta-analyses, approximately 80% of individuals recovering from SARS-CoV-2 infection experience one or more persistent symptoms, highlighting the substantial burden of post-COVID syndrome. The most commonly reported manifestations include fatigue and reduced exercise tolerance (53.5%), exertional dyspnea (51.3%), respiratory symptoms (49.4%), and hair loss (44.1%) [2].

Post-COVID syndrome is of particular clinical importance in individuals with metabolic disorders, especially type 2 diabetes mellitus, which has consistently been associated with an increased risk of severe COVID-19 and adverse clinical outcomes [3,4]. T2DM is characterized by chronic hyperglycemia accompanied by impaired innate immune responses, a pro-inflammatory state, endothelial dysfunction, and alterations in angiotensin-converting enzyme 2 (ACE2) expression [5]. In addition, medications affecting the renin–angiotensin–aldosterone system may further influence the course of SARS-CoV-2 infection, collectively contributing to poorer outcomes and an increased risk of severe complications in patients with COVID-19 [6,7].

Immune dysregulation associated with T2DM may substantially influence the development and clinical manifestations of post-COVID syndrome [8]; however, the underlying mechanisms remain incompletely understood. Although numerous studies have established the association between T2DM and the severity of acute COVID-19, data regarding the clinical and immunological characteristics of post-COVID syndrome in this population remain limited. Furthermore, long-term outcomes may be influenced by multiple factors, including SARS-CoV-2 variants, vaccination status, sex, body mass index, comorbidities, antidiabetic therapy, and individual immune response characteristics [9,10].

Therefore, comprehensive clinical and immunological studies are needed to improve understanding of the mechanisms underlying post-COVID syndrome more than one year after SARS-CoV-2 infection in older adults with T2DM. Such investigations may contribute to the development of more personalized approaches to the long-term management of this vulnerable population.

The aim of the present study was to investigate the clinical, immunological, and metabolic characteristics of post-COVID syndrome in older adults with type 2 diabetes mellitus.

## 2. Materials and Methods

### 2.1. Study Design and Participants

The present study was designed as a cross-sectional descriptive comparative clinical and immunological investigation. It was conducted at the Municipal State-Owned Enter-prise “City Polyclinic No. 4” in Almaty, Kazakhstan, during the period from 2023 to 2024.

The study included older adults aged 60 years and above who had recovered from COVID-19 at least 12 months prior to enrollment. The selection of this age group was based on the higher prevalence and greater clinical severity of post-COVID syndrome among older individuals, as well as the need to assess clinical, immunological, and metabolic features of the post-COVID period in patients with type 2 diabetes mellitus.

A total of 141 patients were enrolled. For each participant, an individual case report form was completed, including data on age, sex, comorbid conditions, COVID-19 vaccination status, current medication use, body mass index, date and severity of the acute SARS-CoV-2 infection, and the presence of clinical manifestations of post-COVID syn-drome.

Participants were selected using a simple random sampling method. Patients with a previously established diagnosis of type 2 diabetes mellitus were assigned to the main group, whereas individuals without disorders of carbohydrate metabolism constituted the comparison group.

Exclusion criteria included age below 60 years, SARS-CoV-2 infection less than 12 months prior to enrollment, decompensated cardiovascular, respiratory, hepatic, or renal diseases, severe cognitive impairment precluding informed consent, and refusal to participate in the study. Previous SARS-CoV-2 infection was confirmed based on clinical his-tory and serological evidence (IgG antibodies), while PCR testing was not consistently available for all patients, as it reflects the acute phase of infection.

Written informed consent was obtained from all participants prior to inclusion. The study protocol was approved by the Ethics Committee of Asfendiyarov Kazakh National Medical University (No. 2 (125), 23 February 2022) and was conducted in accordance with the principles of the Declaration of Helsinki.

### 2.2. Clinical Data Collection

Clinical and anamnestic data were collected during face-to-face examinations at the time of enrollment. All participants underwent a clinical assessment including evaluation of complaints, medical history, and physical examination findings.

The collected information comprised data on clinical manifestations of post-COVID syndrome, comorbidities, ongoing pharmacological therapy, body mass index, and COVID-19 vaccination status. Symptoms of post-COVID syndrome were assessed based on patient-reported complaints and medical record documentation, in accordance with the criteria described in Section 2.3.

### 2.3. Definition of Post-COVID Syndrome

Post-COVID syndrome was defined according to the World Health Organization (WHO) criteria as the presence of symptoms that developed during or after SARS-CoV-2 infection, persisted for at least 12 weeks, and could not be explained by an alternative diagnosis.

Patients were classified as having post-COVID syndrome if they reported one or more of the following symptoms persisting for ≥12 weeks after the acute phase of COVID-19: fatigue, dyspnea, palpitations, cognitive impairment, headache, dizziness, musculoskeletal pain, or other related complaints.

Given the nonspecific nature of many post-COVID symptoms, particularly in older adults with multiple comorbidities, symptoms were considered if they were reported by patients as having appeared after SARS-CoV-2 infection.

### 2.4. Vaccination History and Classification of Participants

Vaccination status was determined according to patient-reported history. Participants were classified as vaccinated if they had received at least one dose of a COVID-19 vaccine before SARS-CoV-2 infection. Because the study was conducted more than one year after infection, detailed information regarding vaccination dates, booster doses, and the exact number of previous SARS-CoV-2 infections was incomplete for some participants. Therefore, analyses were performed according to vaccination status only (vaccinated versus unvaccinated).

### 2.5. Sample Collection and Laboratory Analysis

Venous blood samples were collected from the cubital vein in the morning after an overnight fast, following standard preanalytical requirements. The samples were used for immunological and biochemical analyses.

Quantitative measurement of IgG antibodies to SARS-CoV-2 was performed in a certified clinical laboratory (“3D-Omicron”, Almaty, Kazakhstan) using a commercially available enzyme-linked immunosorbent assay (ELISA) kit (Vector-Best, Novosibirsk, Russia) according to the manufacturer’s instructions. Measurements were conducted using a Stat Fax 2100 microplate reader (Awareness Technology Inc., Palm City, FL, USA). The cut-off value for seropositivity was defined as ≥10 BAU/mL.

Laboratory evaluation also included complete blood count and biochemical analysis. The biochemical parameters comprised total protein, alanine aminotransferase (ALT), aspartate aminotransferase (AST), total bilirubin, urea, creatinine, glucose, total cholesterol, creatine phosphokinase (CPK), lactate dehydrogenase (LDH), and glycated hemoglobin (HbA1c), measured using automated analyzers in accordance with standard laboratory protocols.

### 2.6. Participant Flow

All patients enrolled in the study met the inclusion criteria and had no exclusion criteria. Following initial screening and clinical evaluation, all 141 participants proceeded to the laboratory assessment stage and were included in the final analysis. No participants with incomplete data or withdrawal from the study were identified.

### 2.7. Statistical Analysis

Statistical analysis was performed using IBM SPSS Statistics (IBM Corp., Armonk, NY, USA; version 19). The normality of quantitative variables was assessed using the Shapiro–Wilk test.

Due to non-normal data distribution, results are presented as the median and inter-quartile range (Me [Q1–Q3]). Comparisons between two independent groups were per-formed using the Mann–Whitney U test.

Categorical variables are presented as absolute and relative frequencies (*n*, %). Group differences for categorical variables were evaluated using Pearson’s χ^2^ test, or Fisher’s ex-act test when expected cell counts were less than five. Differences were considered statistically significant at *p* < 0.05.

## 3. Results

### 3.1. Clinical and Demographic Characteristics of the Study Groups

To investigate the clinical and immunological characteristics of older adults with type 2 diabetes mellitus in the long-term period following COVID-19, 141 participants underwent comprehensive clinical and laboratory assessment, including evaluation of humoral immune response parameters. Based on the presence of post-COVID symptoms at the time of assessment, conducted more than one year after SARS-CoV-2 infection, participants were classified into two groups: 75 individuals (53.2%) with post-COVID syndrome and 66 individuals (46.8%) without post-COVID syndrome.

The study population was predominantly female, with women accounting for 88.7% (*n* = 125) of all participants. Most individuals belonged to older age categories: 69.5% (*n* = 98) were classified as older adults and 30.5% (*n* = 43) as being of advanced age. The median age of the cohort was 70 years (66–76 years). T2DM was present in 45.4% of participants (*n* = 64), whereas 54.6% (*n* = 77) had no disorders of glucose metabolism. Among patients with T2DM, the majority were receiving oral glucose-lowering therapy (*n* = 38, 59.4%), while 26 patients (40.6%) were treated with insulin.

**Figure 1:**
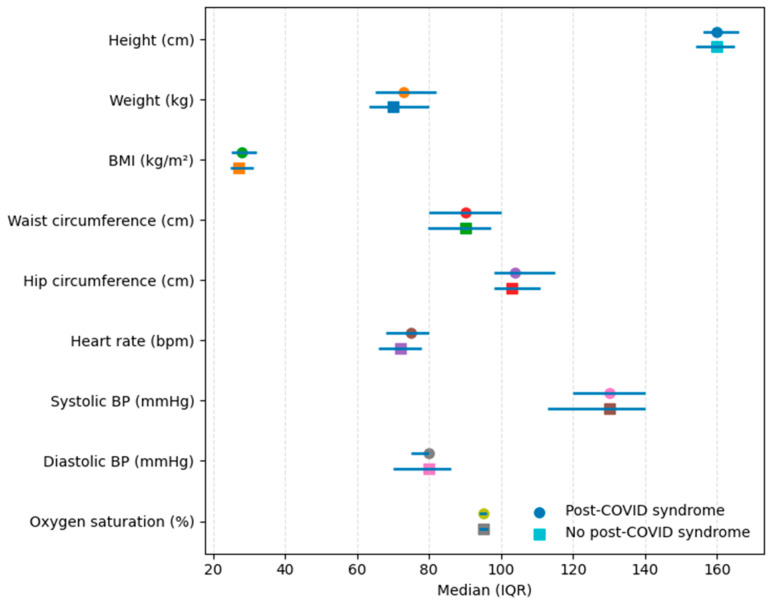
Comparison of anthropometric and physiological parameters in patients with post-COVID syndrome (*n* = 75) and without post-COVID syndrome (*n* = 66). Data are presented as median (Q1–Q3); between-group differences were assessed using the Mann–Whitney U test.

Comparative analysis of anthropometric and physiological characteristics showed no statistically significant differences between participants with and without post-COVID syndrome. Height, body weight, body mass index, waist and hip circumference, heart rate, blood pressure, and peripheral oxygen saturation were comparable between the groups (all *p* > 0.05), indicating a high degree of baseline similarity in physical characteristics (Figure 1).

Among the 75 patients with manifestations of post-COVID syndrome, cardiovascular symptoms were the most prevalent, being identified in 30.7% of cases. This symptom cluster included episodes of palpitations, cardiac rhythm disturbances, sensations of chest pressure or discomfort, as well as features consistent with postural tachycardia.

Asthenic manifestations, primarily represented by generalized weakness as a key feature of asthenic syndrome, were reported by 21.3% of patients. Cognitive disturbances, including impaired concentration, deterioration of short-term memory, and subjective complaints of “brain fog,” were observed with a similar frequency (21.3%).

Neurological symptoms, such as headache, dizziness, and paresthesia, were documented in 20.0% of patients. Osteoarthromyalgic manifestations, characterized by joint and/or muscle pain, were reported by 12.0% of individuals.

**Figure 2:**
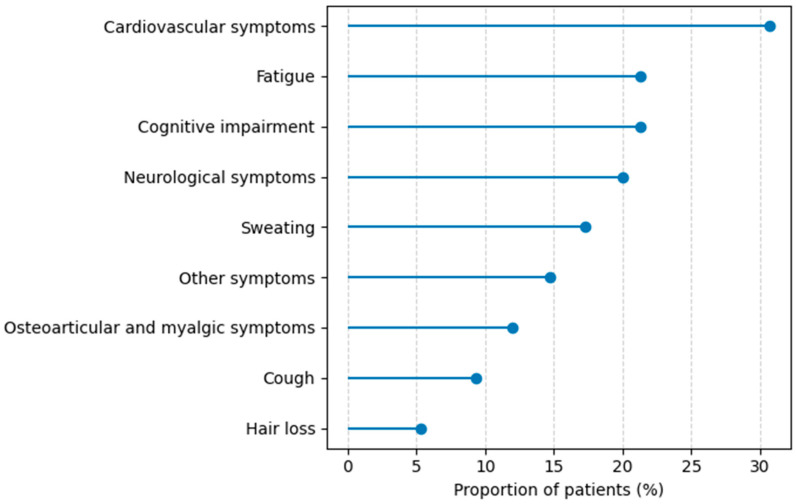
Distribution of clinical manifestations among patients with post-COVID syndrome (*n* = 75). Data are presented as the percentage of patients reporting each symptom at the time of assessment.

Additional symptoms included hyperhidrosis, noted in 17.3% of patients, cough in 9.3%, and hair loss in 5.3% of cases. Furthermore, 14.7% of patients reported various non-specific and less common complaints, which were grouped under the category “other”, which included individual symptoms not attributable to the main clinical clusters (Figure 2).

To explore potential pathophysiological differences between participants with and without post-COVID syndrome, a comparative analysis of laboratory parameters was performed. Evaluation of complete blood count indices and biochemical markers revealed no statistically significant differences between the groups for most of the assessed variables (all *p* > 0.05).

Hemoglobin concentration, erythrocyte, leukocyte, and platelet counts, as well as erythrocyte sedimentation rate (ESR) and leukocyte differential parameters (segmented neutrophils, eosinophils, monocytes, and lymphocytes), were within reference ranges and comparable between participants with and without post-COVID syndrome. The only exception was the percentage of band neutrophils. Although the median values were identical in both groups (1.0% (1.0–1.0)), a statistically significant difference was detected using the Mann–Whitney U test (*p* = 0.033). However, given the absence of observable differences between groups, this finding was considered unlikely to be clinically meaningful and was not interpreted further.

Biochemical parameters, including total protein, urea, creatinine, alanine aminotransferase, aspartate aminotransferase, alkaline phosphatase, creatine phosphokinase, lactate dehydrogenase, glucose, glycated hemoglobin and total cholesterol, also showed no significant differences between the groups (all *p* > 0.05). However, a modest but statistically significant increase in the De Ritis ratio was observed in participants without post-COVID syndrome compared with those with post-COVID syndrome (0.9 (0.8–1.2) vs. 0.8 (0.7–1.1); *p* = 0.019). Although statistically significant, the magnitude of this difference was small and unlikely to be clinically relevant.

SARS-CoV-2-specific IgG antibody levels were comparable between the two groups. Although participants with post-COVID syndrome exhibited numerically higher median IgG concentrations than those without post-COVID syndrome (180.2 (121.8–226.0) vs. 156.0 (126.7–224.0) BAU/mL), the difference did not reach statistical significance (*p* = 0.548), suggesting no significant difference in humoral immune response between the groups.

### 3.2. Clinical and Immunological Differences Between Vaccinated and Unvaccinated Patients

To evaluate the association between COVID-19 vaccination status and laboratory parameters in patients with post-COVID syndrome, a comparative analysis was conducted between vaccinated and unvaccinated individuals. Vaccinated participants were defined as those who had received at least one dose of a COVID-19 vaccine before SARS-CoV-2 infection.

Among the 75 participants with post-COVID syndrome, 49 (65.3%) were unvaccinated against SARS-CoV-2, whereas 26 (34.7%) had received at least one vaccine dose before acute infection. Comparative analysis demonstrated that most hematological and biochemical parameters were within reference ranges and did not differ significantly between the groups.

Complete blood count parameters were comparable between vaccinated and unvaccinated participants. Hemoglobin concentrations were 136 g/L (128–142) and 132 g/L (125–145.5), respectively (*p* = 0.385), while erythrocyte counts were 4.6 × 10^12^/L (4.2–5.0) and 4.5 × 10^12^/L (4.1–5.0), respectively (*p* = 0.604). Leukocyte counts, differential leukocyte parameters, and lymphocyte levels were within physiological ranges and showed no significant differences between groups (all *p* > 0.05). The only statistically significant difference was a higher platelet count among vaccinated participants (285 × 10^9^/L (226–333) vs. 254 × 10^9^/L (215–294); *p* = 0.036).

Biochemical parameters, including total protein, urea, creatinine, total cholesterol, alanine aminotransferase, aspartate aminotransferase, lactate dehydrogenase, creatine phosphokinase, and alkaline phosphatase, did not differ significantly between the groups (all *p* > 0.05) and remained within reference ranges. These findings indicate no clinically relevant differences in hepatic or renal laboratory parameters according to vaccination status. Although AST levels were slightly higher in vaccinated participants (38.8 U/L (27.3–47.9) vs. 29.5 U/L (24.4–38.0); *p* = 0.046), the magnitude of the difference was small and unlikely to be clinically meaningful. Similarly, markers of carbohydrate metabolism, including fasting glucose and glycated hemoglobin, were comparable between groups (*p* = 0.806 and *p* = 0.243, respectively), indicating similar metabolic control.

**Figure 3:**
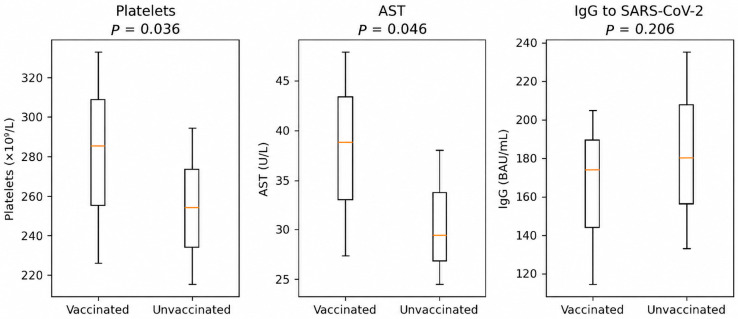
Comparison of platelet count, AST levels, and SARS-CoV-2-specific IgG between vaccinated (*n* = 26) and unvaccinated (*n* = 49) patients with post-COVID syndrome. Data are presented as median (Q1–Q3); between-group differences were assessed using the Mann–Whitney U test.

Analysis of humoral immune response parameters showed that SARS-CoV-2-specific IgG levels were 174 BAU/mL (114–204.7) in vaccinated participants and 180.2 BAU/mL (132.7–235.0) in unvaccinated participants, with no statistically significant difference between the groups (*p* = 0.206). A tendency toward higher IgG levels was observed among unvaccinated individuals, which may reflect the development of a natural humoral immune response following infection. Measurable SARS-CoV-2-specific IgG antibodies remained detectable in vaccinated participants at the time of assessment; however, due to incomplete information regarding vaccination timing and previous SARS-CoV-2 infections, the factors contributing to the observed antibody levels could not be fully evaluated. Figure 3.

### 3.3. Clinical and Immunological Features of Post-COVID Syndrome in Patients with Type 2 Diabetes Mellitus

Among the 75 participants with post-COVID syndrome, 34 (45.3%) had type 2 diabetes mellitus, whereas 41 (54.7%) did not have diabetes. Patients with T2DM tended to have higher body weight, body mass index, waist circumference, and hip circumference than those without diabetes; however, none of these differences reached statistical significance (all *p* > 0.05).

Cardiovascular parameters, including heart rate, blood pressure, and peripheral oxygen saturation, were within normal ranges in both groups and did not differ significantly, indicating comparable cardiorespiratory status at the time of assessment regardless of diabetes status.

Comparative analysis of clinical manifestations demonstrated that most post-COVID symptoms observed more than one year after SARS-CoV-2 infection occurred at similar frequencies in participants with and without T2DM. No statistically significant differences were identified for weakness (*p* = 0.377), hyperhidrosis (*p* = 0.408), cardiovascular symptoms (*p* = 0.733), neurological complaints (*p* = 0.162), cognitive–emotional disturbances (*p* = 0.443), cough (*p* = 0.605), musculoskeletal symptoms (*p* = 0.343), hair loss (*p* = 0.382), or other complaints (*p* = 0.366) (all *p* > 0.05).

**Figure 4:**
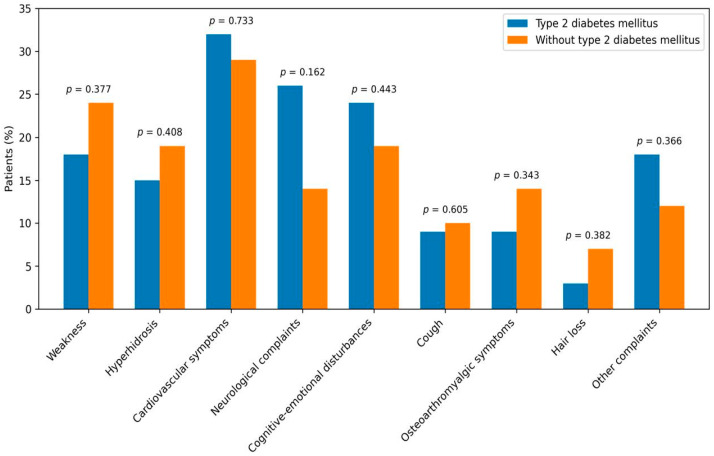
Prevalence of post-COVID syndrome symptoms in patients with post-COVID syndrome, stratified by type 2 diabetes mellitus status: T2DM (*n* = 34) versus non-diabetes (*n* = 41). Data are presented as percentages of patients in each group; between-group comparisons were performed using the χ^2^ test or Fisher’s exact test, as appropriate.

Although patients with T2DM showed a tendency toward a higher overall frequency of reported symptoms, these differences did not reach statistical significance (Figure 4).

Overall, although the differences did not reach statistical significance, patients with type 2 diabetes mellitus tended to exhibit higher body weight, body mass index, waist circumference, and hip circumference than participants without diabetes.

A comparative analysis of hematological and biochemical parameters was performed between patients with previously diagnosed T2DM and non-diabetic individuals who had recovered from COVID-19.

The evaluation of laboratory findings among participants with post-COVID syndrome showed that the presence of T2DM was not associated with significant differences in most hematological parameters. Hemoglobin concentration, erythrocyte, leukocyte, and platelet counts, as well as leukocyte differential parameters, were comparable between the two groups (all *p* > 0.05). Specifically, hemoglobin levels were 134 g/L (126–140) in the T2DM group and 134 g/L (125–145) in the non-diabetic group (*p* = 0.848), while erythrocyte counts were 4.4 × 10^12^/L (4.1–4.9) and 4.6 × 10^12^/L (4.2–5.0), respectively (*p* = 0.628). Similarly, erythrocyte sedimentation rate and platelet counts did not differ significantly between groups (*p* = 0.473 and *p* = 0.721, respectively).

Biochemical analysis demonstrated comparable markers of protein and nitrogen metabolism between participants with and without T2DM. Total protein levels were 74.5 g/L (70.4–79.7) and 75.8 g/L (71.6–80.4), respectively (*p* = 0.584). Urea and creatinine concentrations likewise showed no significant differences (*p* = 0.221 and *p* = 0.778, respectively).

Liver enzyme activity remained within reference ranges in both groups. Alanine aminotransferase levels were 40 U/L (30–46.2) in participants with T2DM and 34.8 U/L (29.8–46.9) in those without diabetes (*p* = 0.492), whereas aspartate aminotransferase levels were 35.6 U/L (27–45) and 30.8 U/L (23.9–39.2), respectively (*p* = 0.084). The De Ritis ratio did not differ significantly between groups (0.9 (0.8–1.0) vs. 0.8 (0.7–1.1); *p* = 0.259). Similarly, total cholesterol, lactate dehydrogenase, creatine phosphokinase, and alkaline phosphatase levels were comparable between groups (all *p* > 0.05).

As expected, parameters of carbohydrate metabolism differed significantly between participants with and without T2DM. Patients with T2DM had higher fasting glucose levels (7.1 mmol/L (6.1–8.0) vs. 5.1 mmol/L (4.7–5.5); *p* = 0.001) and higher HbA1c values (7.0% (6.7–7.9) vs. 6.1% (5.7–6.3); *p* = 0.001). Among participants with T2DM, no statistically significant differences in fasting glucose or HbA1c were observed between those with and without post-COVID syndrome (all *p* > 0.05).

Among participants with post-COVID syndrome, SARS-CoV-2-specific IgG antibody levels were significantly higher in individuals with T2DM than in those without diabetes (193.2 (144.9–262.3) vs. 165.0 (117.2–213.4) BAU/mL; *p* = 0.028). However, among participants with T2DM, IgG levels did not differ significantly between those with and without post-COVID syndrome (193.2 (144.9–262.3) vs. 160.8 (114.4–227.4) BAU/mL; *p* = 0.158). Table 1.

## 4. Discussion

The present study provides a comprehensive assessment of the clinical, metabolic, and humoral immunological characteristics of post-COVID syndrome in older adults evaluated more than one year after SARS-CoV-2 infection, including individuals with type 2 diabetes mellitus and those without disorders of glucose metabolism. The findings demonstrate a high prevalence of symptoms consistent with post-COVID syndrome among participants assessed more than one year after SARS-CoV-2 infection, which is consistent with previous reports [11,12].

**Figure 5:**
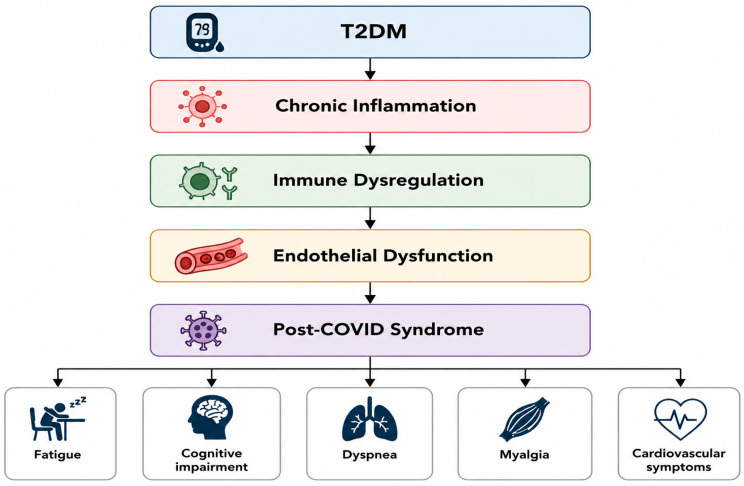
Proposed pathways linking type 2 diabetes mellitus to post-COVID syndrome through chronic inflammation, immune dysregulation, and endothelial dysfunction.

No statistically significant differences in the frequency of post-COVID symptoms were observed between participants with and without T2DM. Nevertheless, the potential contribution of diabetes-related metabolic and vascular alterations, including endothelial dysfunction, microvascular impairment, and chronic low-grade inflammation, to the development and maintenance of post-COVID symptoms has been suggested in previous studies [13,14]. A schematic representation of these proposed mechanisms is presented in Figure 5.

The absence of significant differences in most anthropometric, hematological, and biochemical parameters between participants with and without post-COVID syndrome suggests that systemic inflammatory and metabolic alterations largely resolve during the late post-infectious phase, despite the presence of symptoms at the time of assessment. This observation may be explained by the fact that routine laboratory tests primarily reflect systemic inflammation and general metabolic status, whereas symptoms reported more than one year after SARS-CoV-2 infection may be driven by other mechanisms associated with post-COVID syndrome, including residual immune dysregulation, endothelial and microvascular dysfunction, as well as metabolic and autonomic disturbances that are not fully captured by standard laboratory assessments [15,16].

These findings are consistent with longitudinal studies demonstrating gradual normalization of laboratory parameters within the first year after COVID-19 despite the continued presence of certain clinical manifestations [17]. In contrast, Phetsouphanh et al. reported persistent immune dysregulation and elevated pro-inflammatory mediators during earlier follow-up periods [18]. This discrepancy may be explained by differences in follow-up duration and study populations, including the younger age of participants in the comparison cohort, which may influence recovery trajectories.

Patients with T2DM exhibited significantly higher fasting glucose and glycated hemoglobin levels than those without diabetes, reflecting the underlying metabolic characteristics of this population. These findings suggest that the observed differences in carbohydrate metabolism are more likely attributable to pre-existing metabolic characteristics than to the long-term consequences of SARS-CoV-2 infection.

Elevated SARS-CoV-2–specific IgG levels in participants with T2DM and post-COVID syndrome, compared with individuals without diabetes, may reflect differences in humoral immune responses associated with metabolic dysfunction. However, antibody levels may also be influenced by several factors, including vaccination status, the number of previous SARS-CoV-2 infections, and the time elapsed since the last immunological stimulus. It is well established that antibody titers decline over time, whereas repeated infections or multiple vaccine doses may contribute to higher antibody concentrations [19,20,21]. Therefore, the higher IgG levels observed in participants with T2DM may partly reflect differences in immunological history, including repeated exposure to SARS-CoV-2 or variation in vaccination status. However, detailed information regarding the exact number of previous infections and the timing of vaccination was not available for all participants and therefore could not be formally included in the analysis. In addition, elevated antibody levels may be associated with greater severity of acute COVID-19, as previous studies have demonstrated correlations between the magnitude of the humoral response, disease severity, and inflammatory activity [22,23,24].

The absence of significant differences in humoral immune responses according to vaccination status is consistent with evidence demonstrating the persistence of immunological memory following both vaccination and natural infection over extended periods [25,26]. These findings suggest that humoral immune responses in the late post-COVID period are influenced not only by vaccination status but also by multiple factors, including age, comorbidities, and individual immune characteristics.

Overall, the present findings emphasize the importance of continued clinical assessment of older adults with post-COVID symptoms, even in the presence of largely normalized laboratory parameters. Further longitudinal studies are needed to clarify the long-term clinical significance of the observed metabolic and immunological findings.

However, the present study did not assess cellular immune responses, such as SARS-CoV-2-specific T-cell activity, which may provide additional insights into the immunological mechanisms underlying post-COVID syndrome.

## 5. Conclusions

Post-COVID syndrome symptoms were frequently observed among older adults at the time of assessment, more than one year after SARS-CoV-2 infection, despite normalization of most laboratory parameters.

Patients with type 2 diabetes mellitus exhibited higher fasting glucose, glycated hemoglobin, and SARS-CoV-2-specific IgG antibody levels than individuals without diabetes. These differences are more likely attributable to the underlying metabolic and immunological characteristics of T2DM than to a direct effect of post-COVID syndrome.

The present findings highlight the importance of continued clinical assessment of older adults with post-COVID symptoms, even in the presence of largely normalized laboratory parameters. Further longitudinal studies are needed to clarify the clinical significance of the observed metabolic and immunological findings and to determine optimal follow-up strategies in this population.

## Figures and Tables

**Table 1:** Comparative characteristics of laboratory parameters in patients with post-COVID syndrome with and without type 2 diabetes mellitus.

Parameters	T2DM (*n* = 34) Me(Q2) (Q1–Q3)	Without Diabetes (*n* = 41) Me(Q2) (Q1–Q3)	*p*-Value
Complete Blood Count (CBC)			
Hemoglobin, g/L	134 (126–140)	134 (125–145)	0.848
Erythrocytes, ×10^12^/L	4.4 (4.1–4.9)	4.6 (4.2–5.0)	0.628
Leukocytes, ×10^9^/L	5.4 (4.2–6.3)	5.4 (4.7–6.7)	0.666
Band neutrophils, %	1.0 (1.0–1.0)	1.0 (1.0–1.0)	0.483
Segmented neutrophils, %	59 (53.7–62.2)	60 (54–62.0)	0.814
Eosinophils, %	2.0 (3.0–4.0)	3.0 (3.0–4.0)	0.603
Monocytes, %	3.0 (3.0–3.0)	3.0 (3.0–3.0)	0.070
Lymphocytes, %	33 (30.7–39)	32 (30–38)	0.737
Platelets, ×10^9^/L	253 (217–305)	265 (226–307)	0.721
Erythrocyte sedimentation rate (ESR), mm/h	8.5 (5.0–13.5)	11 (5–15)	0.473
Biochemical Blood Analysis			
Total protein, g/L	74.5 (70.4–79.7)	75.8 (71.6–80.4)	0.584
Urea, mmol/L	5.7 (4.4–6.2)	5.3 (4.3–5.8)	0.221
Creatinine, µmol/L	84.5 (75.3–97.5)	84.8 (77.1–95.6)	0.778
Alanine aminotransferase (ALT), U/L	40 (30–46.2)	34.8 (29.8–46.9)	0.492
Aspartate aminotransferase (AST), U/L	35.6 (27–45.0)	30.8 (23.9–39.2)	0.084
De Ritis ratio (AST/ALT)	0.9 (0.8–1.0)	0.8 (0.7–1.1)	0.259
Total cholesterol, mmol/L	5.2 (4.8–5.7)	5.2 (4.7–5.5)	0.391
Glucose, mmol/L	7.1 (6.1–8.0)	5.1 (4.7–5.5)	0.001
Glycated hemoglobin (HbA1c), %	7.0 (6.7–7.9)	6.1 (5.7–6.3)	0.001
Lactate dehydrogenase (LDH), U/L	351 (253–426)	309 (268–370)	0.363
Creatine phosphokinase (CPK), U/L	169 (144–216)	183 (158–213)	0.257
Alkaline phosphatase (ALP), U/L	227 (208–244.9)	231 (209–239)	0.758
SARS-CoV-2 IgG antibodies			
SARS-CoV-2 IgG antibodies, BAU/mL	193.2 (144.9–262.3)	165 (117–213.4)	**0.028**

*Note.* Statistical significance of differences between groups was assessed using the Mann–Whitney U test. Data are presented as median (Q1–Q3).

## Data Availability

The data supporting the findings of this study are available from the corresponding author upon reasonable request.
